# Supplementary material for: Negative Association of Lignan and Phytosterol Intake with Stress Perception during the COVID-19 Pandemic—A Polish Study on Young Adults
Source: Nutrients. 2024 Feb 2;16(3):445. doi: 10.3390/nu16030445 (PMC10857242; doi:10.3390/nu16030445)
Supplement: Supplementary file 1 [file nutrients-16-00445-s001.zip › nutrients-2824359-supplementary.pdf]

Table S1 Pearson correlation coefficients between Perceived Stress Scale (PSS) and individual items and intake of phytochemicals (N=104).

**Logarithm of daily phytochemical intake**	**PSS**										
	**PSS 1**	**PSS 2**	**PSS 3**	**PSS 4**	**PSS 5**	**PSS 6**	**PSS 7**	**PSS 8**	**PSS 9**	**PSS 10**	**Total**
Total lignans [μg]	-0.289**	-0.327***	-0.229*	-0.043	-0.241*	-0.057	-0.157	-0.256**	-0.109	-0.177	-0.263**
Lariciresinol [μg]	-0.258**	-0.277**	-0.203*	-0.009	-0.258**	-0.088	-0.116	-0.260**	-0.071	-0.149	-0.232*
Matairesinol [ng]	-0.048	-0.113	-0.127	-0.157	-0.029	-0.060	-0.120	-0.090	-0.078	0.067	-0.111
Pinoresinol [μg]	-0.291**	-0.343***	-0.181	-0.026	-0.205*	-0.037	-0.192	-0.227*	-0.133	-0.190	-0.260**
Secoisolariciresinol [μg]	-0.201*	-0.161	-0.272**	-0.158	-0.096	-0.028	-0.057	-0.086	-0.094	-0.010	-0.170
Total phytosterols [mg]	-0.287**	-0.131	-0.197*	-0.125	-0.143	-0.093	-0.261**	-0.120	-0.222*	-0.248*	-0.278**
Stigmasterol [mg]	-0.208*	-0.152	-0.143	-0.271**	-0.217*	-0.079	-0.193*	-0.106	-0.156	-0.223*	-0.255**
Campesterol [mg]	-0.237*	-0.073	-0.145	-0.017	-0.173	-0.082	-0.254**	-0.148	-0.153	-0.227*	-0.231*
β-Sitosterol [mg]	-0.279**	-0.123	-0.184	-0.108	-0.144	-0.069	-0.258**	-0.115	-0.200*	-0.229*	-0.260**

PSS 1. In the last month, how often have you been upset because of something that happened unexpectedly?

PSS 2. In the last month, how often have you felt that you were unable to control the important things in your life?

PSS 3. In the last month, how often have you felt nervous and "stressed"?

PSS 4. In the last month, how often have you felt confident about your ability to handle your personal problems?

PSS 5. In the last month, how often have you felt that things were going your way?

PSS 6. In the last month, how often have you found that you could not cope with all the things that you had to do?

PSS 7. In the last month, how often have you been able to control irritations in your life?

PSS 8. In the last month, how often have you felt that you were on top of things?

PSS 9. In the last month, how often have you been angered because of things that were outside of your control?

PSS 10. In the last month, how often have you felt difficulties were piling up so high that you could not overcome them?

Total. Sum of all points gained in PSS 1-PSS 10 expressed in stens.

*P<0.05, **P<0.01, ***P<0.001 for Student T-test analysis (N=104).

Table S2 Comparison of distribution logarithmically transformed intake of specific phytochemicals between people with normal and elevated perceived stress (N=104).

Logarithm# of daily phytochemical intake	Normal perceived stress (N=52)	Elevated perceived stress (N=52)
Total lignans [μg]	5.31 (1.14)	4.90 (1.15)
Lariciresinol [μg]	4.43 (1.01)	4.11 (1.03)
Pinoresinol [μg]	3.55 (1.59)	2.90 (1.78)
Secoisolariciresinol [μg]	1.46 (1.58)	1.16 (1.34)
Total phytosterols [mg]	8.60 (0.52)	8.34 (0.42)**
Stigmasterol [mg]	5.45 (0.88)	5.08 (0.66)*
Campesterol [mg]	6.06 (0.41)	5.89 (0.48)*
β-sitosterol [mg]	7.88 (0.51)	7.63 (0.43)**

#Logarithm with base 2 was used in transformation, data are presented as Means (SDs), *P<0.05, **P<0.01, ***P<0.001 for Student T-test analysis (N=104).

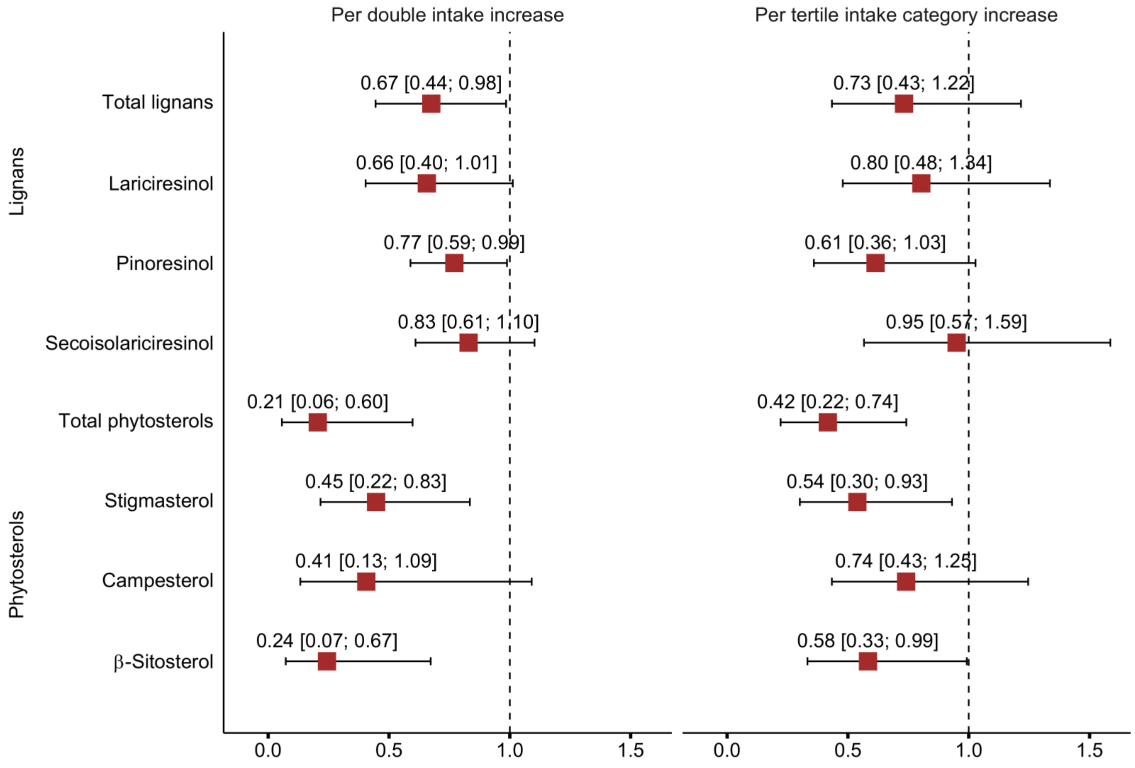

Figure S1. Comparison of odds ratios of elevated perceived stress associated with double increment of phytochemical intake and with increasing tertile category per 1. Results of multiple logistic regression analysis for fully adjusted models after controlling for alcohol drinking, smoking status, BMI; sex, body fat, age and physical activity (N=104).
